# 973 Family-Centered Interdisciplinary Rounds in the Burn Unit

**DOI:** 10.1093/jbcr/iraf019.504

**Published:** 2025-04-01

**Authors:** Shawna Thomas, Caitlin Sayers, Natalie Fitzgerald, Audrey O’Neil, Brett Hartman

**Affiliations:** Eskenazi Health; Eskenazi Health; Richard M. Fairbanks Burn Center; Richard M. Fairbanks Burn Center; Eskenazi Health

## Abstract

**Introduction:**

Multidisciplinary rounds (MDR), a patient-centered care model in which healthcare professionals from varying disciplines collaborate in real time to improve communication between members and optimize patient outcomes. However, the patient’s family may not be included due to challenges including infection rates, pain management, fear of intense reactions to complex wounds, etc. Family-Centered Interdisciplinary Rounds (FCIR) was instituted in this Adult Verified Burn Center as a quality improvement project in May of 2024 to improve communication and patient experience. The goal of this study to evaluate the impact of FCIR on patient experiences and assess any potential complications.

**Methods:**

Historically, MDR occurred on Mondays, Wednesday and Friday mornings between 8:30 and 9:00am. However, hospital visiting hours occur from 12:00pm-8:00pm, which limited family participation in MDR. FCIR was integrated into Monday and Friday morning rounds by allowing family members and loved ones to access the patient room 15 minutes prior to starting rounds. Visitors during rounds donned personal protective equipment (PPE) and instructed not to touch the patient’s wounds. Once the team had entered the patient’s room for assessments, the patient and family were given an opportunity to participate in the team discussion. Infection rates including hand hygiene, CLABSIs, Wound Infection Rates, and patient experience scores were compared prior to and following implementation of the project.

**Results:**

Infection and patient experience data was collected from January through April 2024 to assess outcomes prior to implementation of FCIR, and from May through August 2024 to assess post-implementation outcomes. Following implementation of FCIR, the unit experienced improved hand hygiene with a pre-FCIR average of 85% and a post-FCIR average of 93.5%, Wound Infection pre-FCIR was at 1.29%, which decreased to 0% post-FCIR. CLABSI rates remained at 0% pre and post FCIR implementation. Patient experience scores were assess by quarter. The unit experienced a slight decrease in patient experience, specifically rated to “communication with doctor”, from 99% pre-FCID to 95% post- FCID. However, “communication with nurses” increased from 87% to 97% post- FCID.

**Conclusions:**

Implementation of FCIR improved patient experience when communicating with nurses and did not impact infection rates on the inpatient burn unit. Permitting family presence during MDR provided a direct avenue for family participation in care making decisions. Patient-centered care begins with the patient and their family. Including family presence during important care discussion not only improves communication but can assist in optimizing care outcomes.

**Applicability of Research to Practice:**

Patients and their families are part of the multidisciplinary team and should be allowed to participate in multidisciplinary rounds to optimize outcomes.

**Funding for the Study:**

N/A

